# TIM‐3 as a potential exhaustion marker in CD4^+^ T cells of COVID‐19 patients

**DOI:** 10.1002/iid3.526

**Published:** 2021-09-09

**Authors:** Zahra Modabber, Mehdi Shahbazi, Roghayeh Akbari, Mojgan Bagherzadeh, Alireza Firouzjahi, Mousa Mohammadnia‐Afrouzi

**Affiliations:** ^1^ Student Research Committee Babol University of Medical Sciences Babol Iran; ^2^ Department of Pathology, School of Medicine Babol University of Medical Sciences Babol Iran; ^3^ Immunoregulation Research Center, Health Research Institute Babol University of Medical Sciences Babol Iran; ^4^ Department of Immunology, School of Medicine Babol University of Medical Sciences Babol Iran; ^5^ Department of Internal Medicine, School of Medicine Babol University of Medical Sciences Babol Iran

**Keywords:** CD39, COVID‐19, exhausted Cell, PD‐1, TIM‐3

## Abstract

**Background:**

COVID‐19 causes a range of clinical symptoms from mild to critical and can be life‐threatening. Up to now, it has led to many deaths. We aimed to evaluate exhausted markers on CD4^+^ T cells of COVID‐19 patients.

**Methods:**

In this study, we evaluated 44 patients with confirmed COVID‐19 disease and 16 healthy individuals. Patients were divided into moderate/severe and critical groups. Peripheral blood mononuclear cells (PBMCs) were isolated and stained by anti‐human CD39, PD‐1, TIM‐3, and anti‐human CD4. The percentage of each CD4^+^ subpopulation was calculated by flow cytometry. Furthermore, we collected clinical information and laboratory data of both control and patient groups.

**Results:**

We detected overexpression of TIM‐3 on CD4^+^ T cells in both critical and moderate/severe patients than in healthy individuals (HIs; *p* < .01 and *p* < .0001, respectively). CD4^+^ TIM‐3^+^ CD39^+^ lymphocytes were significantly higher in the critical patients than in HI (*p* < .05). Both Patient groups showed lymphopenia in comparison with HI, but CD4^+^ lymphocytes did not show any significant difference between study subjects. The increased amount of C‐reactive protein, erythrocyte sedimentation rate, creatinine, blood urea nitrogen, and neutrophil count was observed in patients compared to HI.

**Conclusion:**

T cell exhaustion occurs during COVID‐19 disease and TIM‐3 is the most important exhausted marker on CD4^+^ T cells.

## Graphical abstract

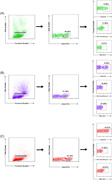

**Abbreviations**
ARDS: acute respiratory distress syndrome
COVID‐19: coronavirus disease‐2019
CRP: C‐reactive protein
CTLA‐4: cytotoxic T lymphocyte antigen‐4
ENTPD1: ectonucleoside triphosphate diphosphohydrolase‐1
ESR: erythrocyte sedimentation rate
G‐CSF: granulocyte colony‐stimulating factor
HRCT: high‐resolution computed tomography
IL‐2: interleukin 2
IP‐10: interferon‐inducible protein 10
LAG3: lymphocyte activation gene 3
LDH: lactate dehydrogenase
MCP‐1: monocyte chemoattractant protein‐1
MIP‐1α: macrophage inflammatory protein‐1 alpha
PBMC: Peripheral blood mononuclear cell
PD‐1: programmed cell death receptor 1
RT‐PCR: reverse transcription‐polymerase chain reaction
SARS‐CoV‐2: severe acute respiratory syndrome coronavirus 2
TIM‐3: T‐cell immunoglobulin and mucin‐domain containing‐3
TNF‐α: tumor necrosis factor alpha

## 1 INTRODUCTION

Coronavirus‐2 is the leading cause of new coronavirus disease‐2019 (COVID‐19), which was first diagnosed in late 2019 (in Wuhan city, Hubei Province, China) in patients with pneumonia of unknown source.^1^ COVID‐19 causes a range of clinical symptoms from mild to critical. Most patients experience mild symptoms in the form of cough, sore throat, and fever. Although many cases experience spontaneous improvement, some patients experience acute respiratory distress syndrome (ARDS) and other lethal complications, such as septic shock, organ failure, pulmonary edema, and severe pneumonia. Additionally, most patients admitted to the intensive care unit (ICU) were older and/or had chronic underlying diseases, such as cardiovascular, respiratory, and cerebrovascular ones, as well as diabetes and hypertension.^1^, ^2^

Lymphopenia has been seen in most patients. In severe cases, cytokine storm was detected as high levels of pro‐inflammatory cytokines, including interleukin 2 (IL‐2), IL‐7, IL‐10, granulocyte colony‐stimulating factor (G‐CSF), interferon‐inducible protein 10 (IP‐10), monocyte chemoattractant protein‐1 (MCP‐1), macrophage inflammatory protein‐1 alpha (MIP‐1A), and tumor necrosis factor alpha (TNF‐α). Thus, it seems that these factors are effective in the pathogenesis of COVID‐19 and lead to severe complications.^3^ The adaptive immune system plays an important role in the immune response and clearance of the virus.^4^ CD4^+^ T cells help B cells produce antibodies, adjust macrophage function, and improve cytotoxic T cells responses. Dysfunction and reduction of CD4^+^ T cells predispose an individual to many infectious diseases.^5^

During cancer together with viral, parasitic, and bacterial infections, progressive dysfunction of T cells with overexpression of immune checkpoint inhibitors (ICIs) appears, which is defined as T cell exhaustion.^6^, ^7^ In many viral infections, increased expression of inhibitory receptors on exhausted T cells, such as programmed cell death receptor 1 (PD‐1), T‐cell immunoglobulin and mucin‐domain containing‐3 (TIM‐3), cytotoxic T lymphocyte antigen‐4 (CTLA‐4), CD39 (ectonucleoside triphosphate diphosphohydrolase‐1 [ENTPD1]), and lymphocyte activation gene 3 (LAG3) were detected, which resulted in T cell hyporesponsiveness. Exhausted T cells have dysfunction and cannot respond to viruses or eliminate them.^8^, ^9^, ^10^ Therefore, in addition to lymphopenia, lymphocyte dysfunction also appears to be effective in disease pathogenesis. It seems that increasing the number and function of T helper (Th) lymphocytes can be useful in disease management. Until now, little research has been done on the exhaustion of T cells in COVID‐19 patients. Thus, this study aims to evaluate the exhausted markers on CD4^+^ T cells of COVID‐19 patients.

## 2 MATERIALS AND METHODS

### 2.1 Study population

In this study, we evaluated suspected patients with usual clinical symptoms and characteristic findings in lung high‐resolution computed tomography (HRCT), who were admitted to the Ayatollah Rouhani, Shahid Yahyanezhad, and Shahid Beheshti hospitals, all associated with Babol University of Medical Sciences. Forty‐four patients with a confirmed diagnosis of severe acute respiratory syndrome coronavirus 2 (SARS‐CoV‐2) using quantitative reverse transcription‐polymerase chain reaction (RT‐PCR) assay on throat swab specimen were enrolled in this study. Sixteen healthy individuals (HIs) with negative RT‐PCR were selected as the control group.

Patients were divided into two groups, that is, moderate/severe (who required inpatient care) and critical (who required ICU care) groups according to the World Health Organization (WHO) classification.^11^ The mean length of hospital stay was 24.55 days for critical and 14.11 days for moderate/severe patients. The combination of the drugs including azithromycin, dexamethasone, prednisolone, hydroxychloroquine sulfate, hydrocortisone, and methylprednisolone was used for the treatment of the patients. From all patients, 7 ml of venous blood samples was collected into green‐top tubes (tubes containing sodium heparin).

Clinical information and laboratory data, including complete blood count with differential, biochemistry, C‐reactive protein (CRP), ferritin, erythrocyte sedimentation rate (ESR), and lactate dehydrogenase (LDH), were collected from electronic and paper medical records.

### 2.2 Ethical statement

Written informed consent was received from all study individuals, and the Local Ethical Committee of Babol University of Medical Sciences authorized the study procedure.

### 2.3 Peripheral blood mononuclear cell isolation and flow cytometry analysis

Peripheral blood mononuclear cells (PBMCs) were isolated by the Ficoll‐Hypaque density gradient centrifugation method. The plasma was removed from whole blood by centrifuging for 20 min at 400 g, and a buffy coat ring was accumulated. PBMCs were enumerated in the Neubauer plate after washing in phosphate‐buffered saline (PBS). FACS buffer (PBS containing 0.5% bovine serum albumin) was used to wash 10^6^ cells of isolated PBMCs. Then, PBMCs were treated with FcR blocker (BioLegend). The cells were incubated with PE anti‐human CD39 (Clone A1), PD‐1‐ PerCp‐Cy5.5 Ab (Clone EH12.2H7), TIM‐3 APC‐conjugated Ab (Clone F38‐2E2) (all from BioLegend), and FITC‐labeled anti‐human CD4 (BD Biosciences) in the dark room. FACSCalibur (BD Biosciences) flow cytometer was applied, and FlowJo 7.6.1 for Windows was used for results analysis. The percentage of each CD4^+^ subpopulation was calculated after cell gating by their forward and side scatters parameters.

### 2.4 Statistical analysis

Statistical analysis was accomplished by GraphPad Prism 7.00 for Windows (GraphPad Software), and data were expressed as mean ± standard deviation (SD). Differences in the variables between groups were determined by using an independent sample *t*‐test. The normality of the data was calculated using the Kolmogorov–Smirnov test. *P*‐values <  0.05 were considered significant.

## 3 RESULTS

### 3.1 Demographic and clinical characteristics of study subjects

The demographic and clinical characteristics of the study subjects are shown in Table 1. The patients are age‐ and sex‐matched. Patients had an increased amount of CRP, ESR, creatinine, and blood urea nitrogen compared to HI. Moreover, patients showed a high amount of neutrophil compared with HI. In contrast, lymphocytes were lower in the patients than in HI (Table 1).

**Table 1:** Demographic and clinical characteristics of study subjects

	Healthy (16)	Moderate/severe (30)	Critical (14)	*p*
**Age (years)**	38.19 (8.81)	58.33 (18.49)	52.64 (17.83)	
**Gender**				
Female	5	14	6	
Male	11	16	8	
**Temperature**	36.56 (0.108)	37.41 (1.007)	37.41 (0.401)	<.0001
**Cough**	0 (0%)	23 (76%)	12 (85%)	<.0001
**Smoking**	1 (0.06%)	4 (0.13%)	0 (0%)	.3555
**chronic underlying disease**	0	12 (40%)	10 (71%)	.0006
**LDH (U/L)**	251.9 (80.86)	556.4 (268.5)	1863 (2709)	<.0001
**C‐reactive protein (mg/dl)**	1.746 (1.563)	81.97 (68.83)	191.3 (116.6)	<.0001
**ESR (mm/h)**	10.71 (3.989)	61.21 (45.62)	69.58 (32.49)	<.0001
**Ferritin (ng/ml)**	102.9 (51.98)	689.6 (731.9)	1129 (1071)	.0002
**BUN (mg/dl)**	15.00 (3.486)	21.35 (19.54)	39.54(27.20)	.0005
**Creatinine (mg/dl)**	0.8143 (0.1076)	1.993 (2.764)	2.106 (2.114)	.0673
**AST (IU/L)**	21.36 (3.875)	51.57 (65.96)	120.3 (201.5)	.0100
**ALT (IU/L)**	18.00 (6.493)	39.33 (27.49)	180.5 (333.9)	.0171
**WBC**	7490 (1798)	8243 (3631)	9407 (3693)	.2949
**Lymphocytes (% in differential)**	32.19 (4.324)	23.43 (12.01)	12.29 (5.121)	<.0001
**Lymphocytes (absolute number)**	2384 (534.7)	1825 (1130)	1069 (409.9)	<.0001
**Neutrophil (% in differential)**	67.20 (10.11)	70.07 (11.92)	84.21 (8.059)	.0002
**Neutrophil (absolute number)**	5059 (1530)	5897 (3048)	8036 (3595)	.0234
**PLT**	269,267 (103,425)	267,467 (137,550)	211,429 (132,324)	.3101
**NLR**	2.159 (0.5858)	3.98 (2.308)	8.571 (4.927)	<.0001
**PLR**	114.2 (39.30)	184.4 (108.1)	207.2 (125.9)	.0308
**Hb (mg/dl)**	14.58 (1.546)	11.06 (1.284)	10.45 (1.599)	<.0001

Abbreviations: ALT, alanine aminotransferease; AST, aspartate aminotransferase; BUN, blood urea nitrogen; ESR, erythrocyte sedimentation rate; LDH, lactate dehydrogenase; NLR, neutrophil‐lymphocyte ratio; PLR, platelet‐lymphocyte ratio; PLT, platelet; WBC, white blood cell.

### 3.2 TIM‐3 is highly expressed on CD4^+^ lymphocytes of patients

**Figure 1:**
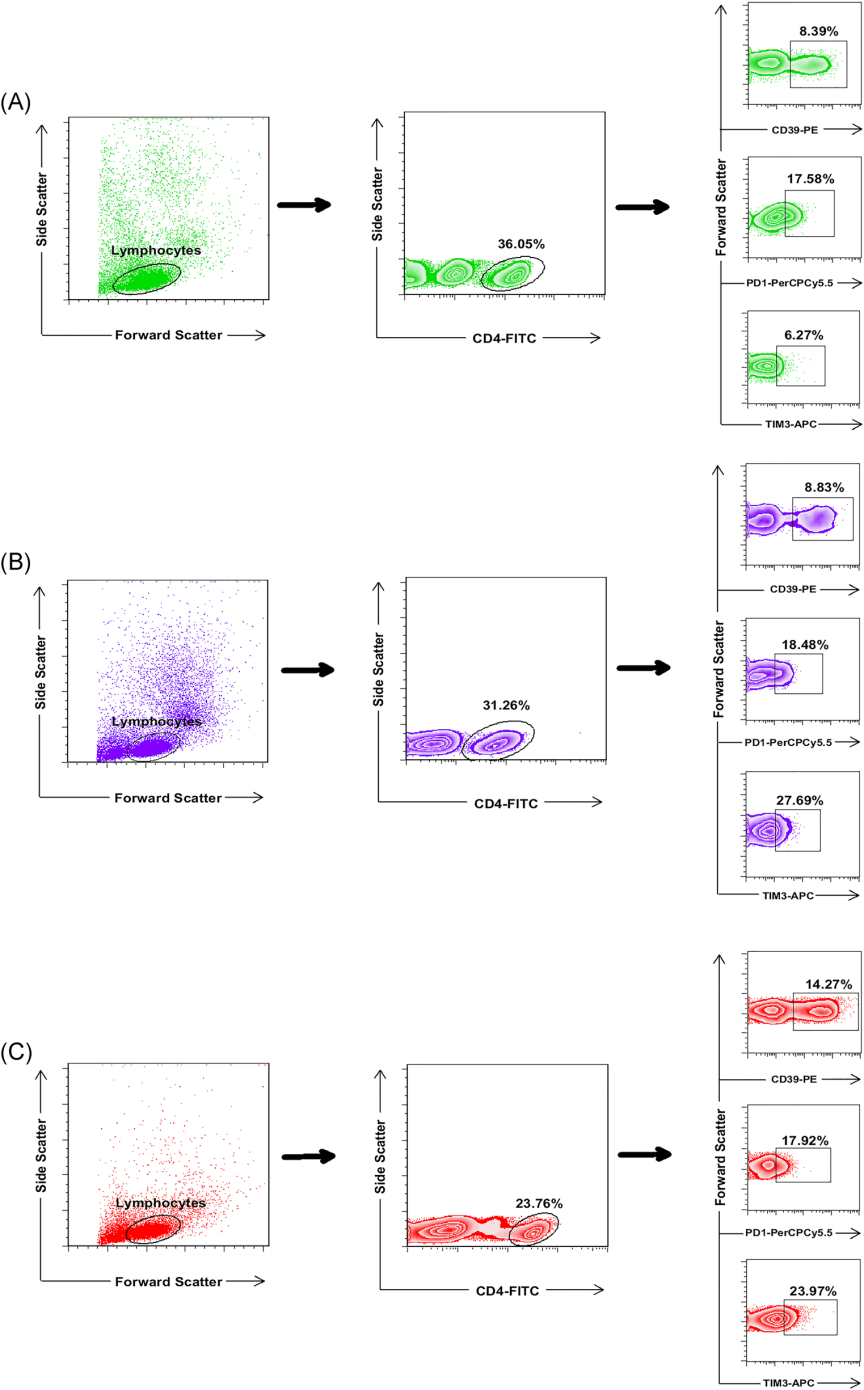
The frequency of CD4^+^ TIM‐3^+ ^lymphocytes was significantly higher in both patient groups. PBMCs were stained with CD4‐FITC, PD‐1‐PerCp‐Cy5.5, CD39‐PE, and TIM‐3‐APC conjugated monoclonal antibodies and analyzed with BD FACSCalibur flow cytometer. Lymphocytes were gated based on forward and side scatters; the frequency of CD39^+^, TIM‐3^+^, and PD‐1^+^ subsets in CD4^+^ T cells, were then detected according to the antibody staining. The frequency of CD4^+^ TIM‐3^+^ lymphocytes was significantly higher in both moderate/severe and critical patients compared to healthy individuals (*p* < .0001 and *p* < .01, respectively). (A) the healthy individuals; (B) the moderate/severe patients; (C) the critical patients. PBMC, peripheral blood mononuclear cell

TIM‐3, PD‐1, and CD39 as the exhausted marker of T cells were evaluated on the CD4^+^ lymphocytes of the study subject. First, the frequency of CD4^+^ TIM‐3^+^, CD4^+^ PD‐1^+^, and CD4^+^ CD39^+^ lymphocytes was evaluated in the subjects. The frequency of CD4^+^ TIM‐3^+^ lymphocytes was significantly higher in both critical and moderate/severe patients than in HI (*p* < .01 and *p* < .0001, respectively; see Table 2). In contrast, there was no significant difference between patients and HI regarding the frequency of CD4^+^ PD‐1^+^ and CD4^+^ CD39^+^ lymphocytes (Table 2 and Figures 1 and 3).

**Table 2:** Frequency of exhausted CD4^+ ^T cells in the subjects

	Healthy (16)	Moderate/severe (30)	Critical (14)	*P‐*value
CD4^+^ (% Lymphocytes)	36.05 (5.676)	31.27 (15.93)	23.76 (13.34)	.0816
CD4^+^ TIM3^+^ (% CD4^+^)	6.270 (5.169)	27.69 (13.57)	23.97 (14.88)	<.0001
CD4^+^ PD1^+^ (% CD4^+^)	17.59 (8.027)	18.48 (8.946)	17.93 (9.164)	.8585
CD4^+^ CD39^+^ (% CD4^+^)	8.393 (2.607)	8.835 (4.887)	14.28 (9.608)	.1173
CD4^+^ TIM3^+^ CD39^+^ (% CD4^+^)	3.065 (2.536)	3.742 (1.935)	5.660 (3.169)	.0304
CD4^+^ TIM3^+^ PD1^+^ (% CD4^+^)	3.375 (2.869)	5.711 (3.549)	4.910 (3.304)	.0727
CD4^+^ PD1^+^ CD39^+^ (% CD4^+^)	4.572 (1.640)	4.612 (2.718)	5.176 (2.638)	.8639
CD4^+^ TIM3^+^ PD1^+^ CD39+ (% CD4^+^)	1.361 (1.019)	1.577 (0.9376)	1.795 (1.469)	.5616

### 3.3 Higher frequency of CD4^+^ TIM3^+^ CD39^+^ lymphocytes in critical patients

**Figure 2:**
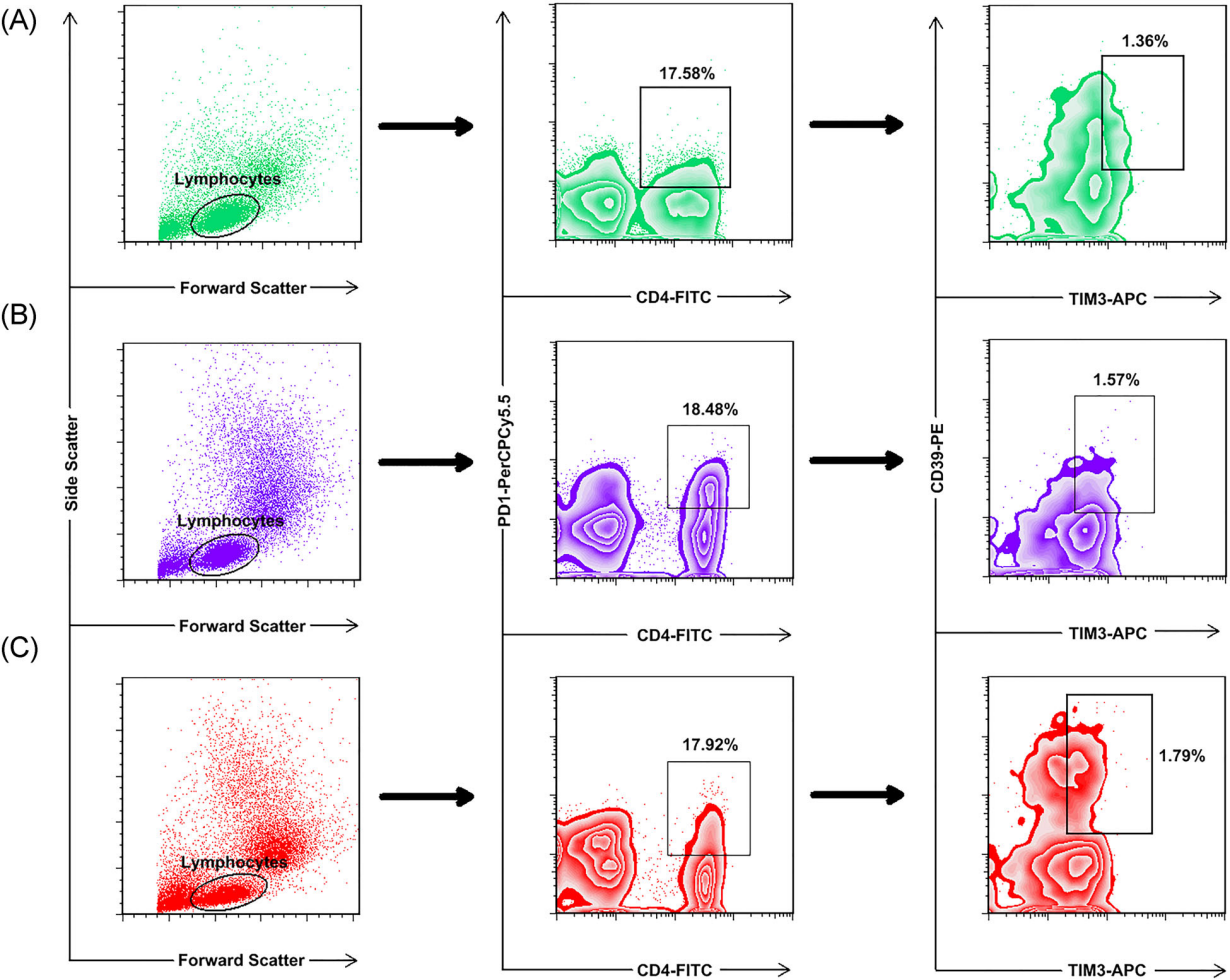
The simultaneous expression levels of TIM‐3 and CD39 had no significant difference in PD‐1^+^ CD4^+^ cells of COVID‐19 patients and healthy individuals. PBMCs were stained with CD4‐FITC, PD‐1‐PerCp‐Cy5.5, CD39‐PE, and TIM‐3‐APC conjugated monoclonal antibodies and analyzed with BD FACSCalibur flow cytometer. PD‐1^+^ CD4^+^ T cells were initially gated from the lymphocyte population to analyze the obtained graphs, and then the simultaneous expression levels of CD39 and TIM‐3 markers were determined in the PD‐1^+^ CD4^+^ T cell populations. (A) the healthy individuals; (B) the moderate/severe patients; (C) the critical patients. PBMC, peripheral blood mononuclear cell

**Figure 3:**
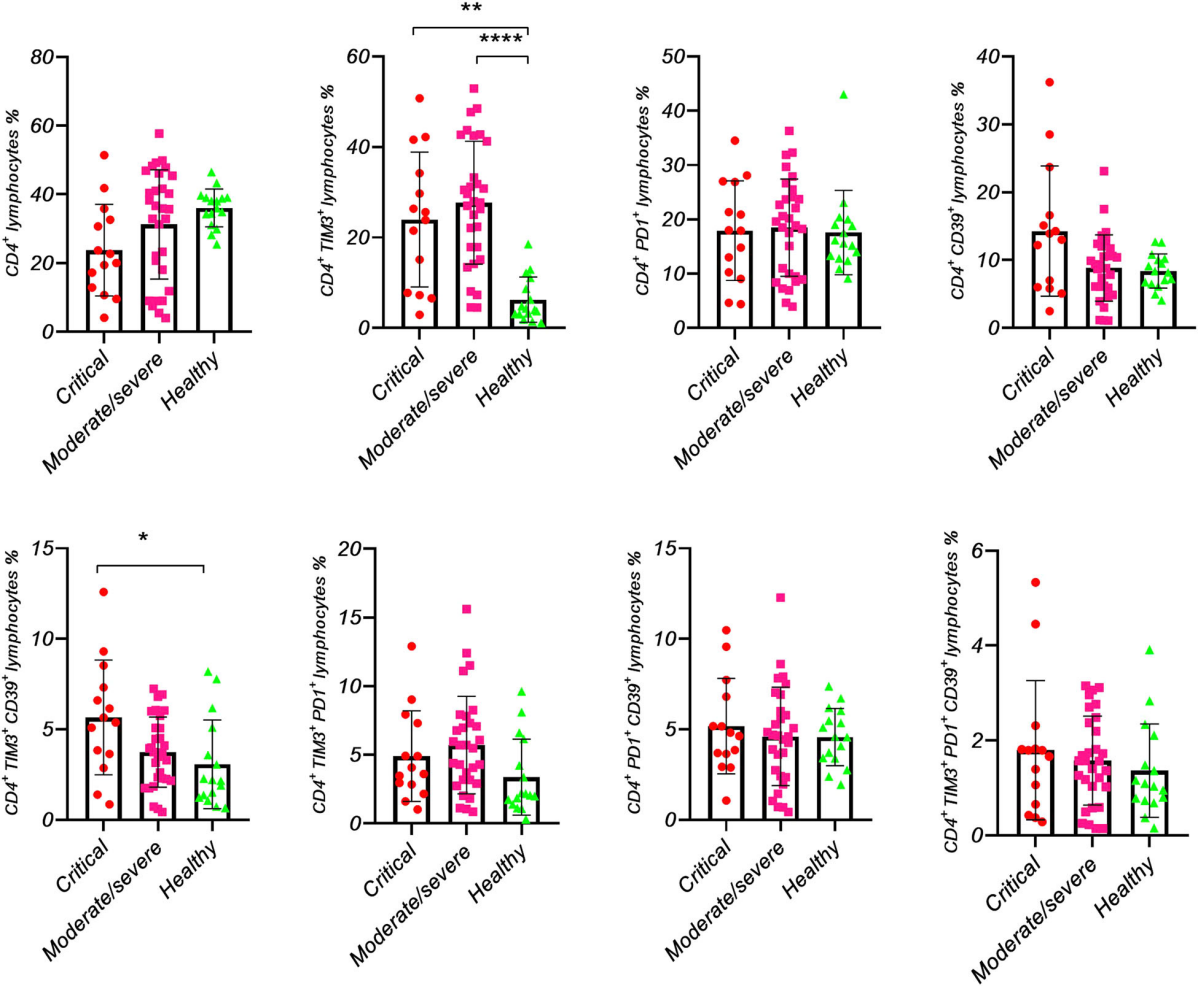
The subset analysis of the exhausted CD4^+ ^cells in healthy individuals and COVID‐19 patients. The percentage of each subset was evaluated in CD4^+^ cells. Each dot represents single healthy individuals or patients. *p* < .05 was considered statistically significant

Simultaneous expression of TIM‐3 CD39, TIM‐3 PD‐1, and PD‐1 CD39 on CD4^+^ lymphocytes was evaluated in the subjects. CD4^+^ TIM‐3^+^ CD39^+^ lymphocytes were significantly higher in the critical patients than in HI (*p* < .05), and no significant difference was observed between critical and moderate/severe patients or moderate/severe and HI. Moreover, there was no significant difference between study subjects regarding the frequency of CD4^+^ TIM‐3^+^ PD‐1^+^ and CD4^+^ PD‐1^+^ CD39^+^ lymphocytes (Table 2 and Figures 2 and 3).

### 3.4 Same frequency of CD4^+^ lymphocytes between subjects

Although patients showed lymphopenia compared to HI, CD4^+^ lymphocytes did not show any significant difference between study subjects (Table 2 and Figure 1). Moreover, when TIM‐3, PD‐1, and CD39 markers were simultaneously analyzed on CD4^+^ lymphocytes, no significant difference was observed between patients and HI (Table 2 and Figure 3).

## 4 DISCUSSION

T cells migration out of blood vessels into interstitial lung tissue result in peripheral T cell lymphopenia.^12^ Similar to other studies, we observed lymphopenia in COVID‐19 patients, which increased with disease progression.^3^, ^13^, ^14^ CD4^+^ T cells have an important role in the immune response by stimulating B cells and cytotoxic T cells.^15^ Activation, expansion, and maintenance of CD8^+^ T cells are affected by CD4^+^ T cells.^16^ Previous studies have shown that CD4^+^ T cells are decreased in patients, particularly in severe ones.^17^, ^18^, ^19^

In this study, total lymphocytes were remarkably decreased in moderate/severe and critical COVID‐19 patients compared with HI (Table 1). However, regarding the frequency of CD4^+^ lymphocytes, no significant difference was observed in all groups (Figure 3). In contrast, in our previous paper, we showed that, besides the total lymphocytes, CD8^+^ lymphocytes were significantly decreased in both ICU and non‐ICU COVID‐19 patients compared with HI (unpublished paper).

There are several negative regulatory pathways involving in T cell exhaustion. During some viral infectious diseases, exhausted CD4^+^ Tcells reveal diminished proliferative capability and lack of multifunctional cytokine response, particularly decreased production of IL‐2, which results in disease progression. The antibody‐mediated blockade of inhibitory receptors improves the functional and proliferative capabilities of exhausted T cells.^6^ Yang et al.^20^ found that the expression of PD‐1 was upregulated in CD4^+^ T cells of COVID‐19 patients. In a study conducted by Diao et al.,^17^ PD‐1 expression in CD4^+^ T cells was also upregulated in COVID‐19 patients and influenced by disease severity. In our experience, PD‐1 overexpression in CD4^+^ T cells of COVID‐19 patients was not observed. However, the expression of TIM‐3 was upregulated.

TIM‐3 is another marker of exhausted T cells. In different settings, it has discrete ligands, which explain its different functions.^21^ Galectin‐9 (Gal‐9) is one of the TIM‐3 ligands. Previous studies have demonstrated that interaction between TIM‐3/Gal‐9 induces apoptosis of Th1 cells.^22^, ^23^ Previous studies on several viral infections have revealed overexpression of TIM‐3 on exhausted T cells due to sustained stimulation.^6^, ^24^, ^25^ In our previous experience, we showed that TIM‐3 was overexpressed by CD8^+^ lymphocytes of patients with active chronic hepatitis B and not with inactive chronic hepatitis B.^26^ Moreover, in our recent study, critical COVID‐19 patients had increased frequency of CD8^+^ TIM‐3^+^ and CD8^+^ TIM‐3^+^ CD39^+^ lymphocytes compared with noncritical and HI subjects (unpublished paper).

Diao et al.^17^ found increased expression of TIM‐3 on CD4^+^ T cells in COVID‐19 patients admitted to the ICU. Consistent with their study, we found increased expression of TIM‐3 on CD4^+^ T cells in both moderate/severe and critical patients. Our paper indicated that CD4^+^ TIM‐3^+^ lymphocytes were remarkably higher in the critical COVID‐19 patients than in the moderate/severe and HI subjects. Thus, our study can support the role of TIM‐3 in Th cell exhaustion during the SARS‐CoV‐2 infection. It has been shown that TIM‐3 blockade immunotherapy can improve the function of exhausted T cells.^27^

CD39 is also associated with T cell exhaustion.^28^ CD39 is an ectoenzyme producing extracellular adenosine by hydrolase of extracellular adenosine triphosphate (ATP). Injured cells secrete extracellular ATP, resulting in pro‐inflammatory responses. On the other hand, extracellular adenosine restricts inflammatory responses by hampering activated immune cells.^29^, ^30^

Bono et al.^29^ reported that CD39 expression was detected in all Th subpopulations. Overexpression of CD39 on T cells was identified during several viral and bacterial diseases.^9^, ^31^, ^32^, ^33^ In this study, we also showed that critical COVID‐19 patients had increased frequency of CD4^+^ TIM‐3^+^ CD39^+^ lymphocytes compared with moderate/severe and HI subjects.

## 5 CONCLUSION

Critical COVID‐19 patients have various dysregulation in their immune system response, such as lymphopenia, cytokine storm, and increased frequency of exhausted CD4^+^ and CD8^+^ lymphocytes. These characteristics consequently could increase the mortality rate, especially in critical patients. In this regard, an attempt to prevent exhaustion of both CD4^+^ and CD8^+^ lymphocytes may help the COVID‐19 patients generate a better response against the disease. As lymphopenia is an indispensable characteristic of CVID‐19 patients, it is crucial to maintain those residual lymphocytes vigorously.

## CONFLICT OF INTERESTS

The authors declare that there are no conflict of interests.

## AUTHOR CONTRIBUTIONS

Zahra Modabber, Roghayeh Akbari, and Mojgan Bagherzadeh drafted the manuscript. Mehdi Shahbazi, Zahra Modabber, Alireza Firouzjahi, and Mousa Mohammadnia‐Afrouzi performed the experiments. Alireza Firouzjahi and Mousa Mohammadnia‐Afrouzi supervised the experiments. All authors participated in data analysis and revision of the manuscript and approved the final version.

## Data Availability

The data that support the findings of this study are available on request from the corresponding author. The data are not publicly available due to privacy or ethical restrictions.
